# Rhesus monkeys exhibiting spontaneous ritualistic behaviors resembling obsessive-compulsive disorder

**DOI:** 10.1093/nsr/nwad312

**Published:** 2023-12-08

**Authors:** Rongwei Zhai, Geya Tong, Zheqin Li, Weichen Song, Yang Hu, Sha Xu, Qiqi Wei, Xiaocheng Zhang, Yi Li, Bingbing Liao, Chenyu Yuan, Yinqing Fan, Ge Song, Yinyin Ouyang, Wenxuan Zhang, Yaqiu Tang, Minghui Jin, Yuxian Zhang, He Li, Zhi Yang, Guan Ning Lin, Dan J Stein, Zhi-Qi Xiong, Zhen Wang

**Affiliations:** Shanghai Mental Health Center, Shanghai Jiao Tong University School of Medicine, Shanghai 200030, China; Institute of Neuroscience, State Key Laboratory of Neuroscience, CAS Center for Excellence in Brain Science and Intelligence Technology, Chinese Academy of Sciences, Shanghai 200031, China; Lingang Laboratory, Shanghai 200031, China; Shanghai Center for Brain Science and Brain-Inspired Technology, Shanghai 201602, China; Shanghai Mental Health Center, Shanghai Jiao Tong University School of Medicine, Shanghai 200030, China; Shanghai Mental Health Center, Shanghai Jiao Tong University School of Medicine, Shanghai 200030, China; Shanghai Mental Health Center, Shanghai Jiao Tong University School of Medicine, Shanghai 200030, China; Shanghai Mental Health Center, Shanghai Jiao Tong University School of Medicine, Shanghai 200030, China; Institute of Neuroscience, State Key Laboratory of Neuroscience, CAS Center for Excellence in Brain Science and Intelligence Technology, Chinese Academy of Sciences, Shanghai 200031, China; Lingang Laboratory, Shanghai 200031, China; Institute of Neuroscience, State Key Laboratory of Neuroscience, CAS Center for Excellence in Brain Science and Intelligence Technology, Chinese Academy of Sciences, Shanghai 200031, China; Lingang Laboratory, Shanghai 200031, China; Institute of Neuroscience, State Key Laboratory of Neuroscience, CAS Center for Excellence in Brain Science and Intelligence Technology, Chinese Academy of Sciences, Shanghai 200031, China; Lingang Laboratory, Shanghai 200031, China; Shanghai Mental Health Center, Shanghai Jiao Tong University School of Medicine, Shanghai 200030, China; Shanghai Mental Health Center, Shanghai Jiao Tong University School of Medicine, Shanghai 200030, China; Shanghai Mental Health Center, Shanghai Jiao Tong University School of Medicine, Shanghai 200030, China; Shanghai Mental Health Center, Shanghai Jiao Tong University School of Medicine, Shanghai 200030, China; Shanghai Mental Health Center, Shanghai Jiao Tong University School of Medicine, Shanghai 200030, China; Shanghai Mental Health Center, Shanghai Jiao Tong University School of Medicine, Shanghai 200030, China; Shanghai Mental Health Center, Shanghai Jiao Tong University School of Medicine, Shanghai 200030, China; Shanghai Mental Health Center, Shanghai Jiao Tong University School of Medicine, Shanghai 200030, China; Shanghai Mental Health Center, Shanghai Jiao Tong University School of Medicine, Shanghai 200030, China; Institute of Neuroscience, State Key Laboratory of Neuroscience, CAS Center for Excellence in Brain Science and Intelligence Technology, Chinese Academy of Sciences, Shanghai 200031, China; Institute of Neuroscience, State Key Laboratory of Neuroscience, CAS Center for Excellence in Brain Science and Intelligence Technology, Chinese Academy of Sciences, Shanghai 200031, China; Shanghai Mental Health Center, Shanghai Jiao Tong University School of Medicine, Shanghai 200030, China; Shanghai Mental Health Center, Shanghai Jiao Tong University School of Medicine, Shanghai 200030, China; Translational Neuropsychiatry Unit (TNU), Department of Clinical Medicine, Aarhus University, Aarhus 8200, Denmark; Institute of Neuroscience, State Key Laboratory of Neuroscience, CAS Center for Excellence in Brain Science and Intelligence Technology, Chinese Academy of Sciences, Shanghai 200031, China; Shanghai Center for Brain Science and Brain-Inspired Technology, Shanghai 201602, China; Shanghai Mental Health Center, Shanghai Jiao Tong University School of Medicine, Shanghai 200030, China; Shanghai Key Laboratory of Psychotic Disorders, Shanghai Mental Health Center, Shanghai Jiao Tong University School of Medicine, Shanghai 200030, China

**Keywords:** obsessive-compulsive disorder (OCD), primate ritualistic behavior model, OCD risk genes, caudate nucleus, fluoxetine

## Abstract

Obsessive-compulsive disorder (OCD) is a chronic and debilitating psychiatric disorder that affects ∼2%–3% of the population globally. Studying spontaneous OCD-like behaviors in non-human primates may improve our understanding of the disorder. In large rhesus monkey colonies, we found 10 monkeys spontaneously exhibiting persistent sequential motor behaviors (SMBs) in individual-specific sequences that were repetitive, time-consuming and stable over prolonged periods. Genetic analysis revealed severely damaging mutations in genes associated with OCD risk in humans. Brain imaging showed that monkeys with SMBs had larger gray matter (GM) volumes in the left caudate nucleus and lower fractional anisotropy of the corpus callosum. The GM volume of the left caudate nucleus correlated positively with the daily duration of SMBs. Notably, exposure to a stressor (human presence) significantly increased SMBs. In addition, fluoxetine, a serotonergic medication commonly used for OCD, decreased SMBs in these monkeys. These findings provide a novel foundation for developing better understanding and treatment of OCD.

## INTRODUCTION

Obsessive-compulsive disorder (OCD) is a chronic and debilitating psychiatric disorder characterized by the presence of recurrent intrusive thoughts (obsessions) or repetitive ritualistic behaviors (compulsions), or both in most cases [1]. The lifetime prevalence rate of OCD in the general population is estimated to be 2%–3% [2]. However, ∼40% of OCD patients do not respond to currently available treatments [3–5], posing significant challenges for OCD therapy and research.

Individuals with OCD often exhibit ritualistic behaviors that are highly reminiscent of fixed sequential motor behaviors (SMBs) in other species [6]. Non-human primates (NHPs) have emerged as an ideal system for comprehensively studying these complex behaviors [7,8] and the cognitive-motor dysfunctions characteristic of disorders like OCD [9,10]. NHPs possess diverse behavioral repertoires, emotional complexity and cognitive abilities [11,12], making them highly suitable for conducting research in this field. Furthermore, their evolutionary proximity to humans [13,14] facilitates the translation of findings from NHP studies, including insights into disease mechanisms and intervention strategies, to the human context. Therefore, NHPs may serve as a particularly appropriate animal model for studying the intricate ritualistic behaviors associated with OCD.

A subset of single-caged monkeys exhibits multiple spontaneous, stereotypical motor behaviors [15]. If these behaviors occur in a fixed sequence repetitively, they may constitute an experimental model for investigating compulsive ritualistic behaviors in humans. In this study, we identified 10 rhesus monkeys (*Macaca mulatta*) with persistent repetitive SMBs among large colonies of cage-reared rhesus monkeys. Further analysis of the daily pattern and stability of SMBs in these monkeys revealed similarities in phenomenology to rituals seen in humans with OCD [6], suggesting similar genetic and neural mechanisms underlying monkey SMBs and human OCD. Genetic analysis indeed revealed severely damaging mutations in candidate risk genes for OCD in humans, suggesting a potential genetic contribution to SMB pathology in these monkeys. Furthermore, magnetic resonance imaging (MRI) studies revealed structural abnormalities in the cortico-striatal system and corpus callosum of these monkeys, in line with MRI findings in humans with OCD. Additionally, exposure to a stressor (the presence of humans) led to a significant increase in SMBs. Finally, we found that the administration of fluoxetine, a commonly used OCD drug, resulted in partial alleviation of SMBs in these monkeys. These findings provide a novel foundation for understanding OCD pathology and developing more effective treatments for this condition.

## RESULTS

### Spontaneous and persistent SMBs in single-caged rhesus monkeys

**Figure 1.**
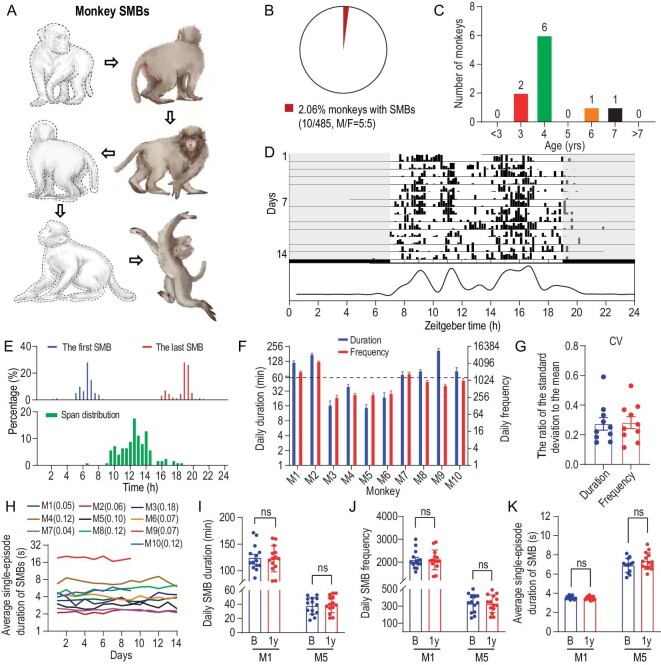
Occurrences, daily patterns and stability of sequential motor behaviors (SMBs) in rhesus monkeys. (A) Schematic diagram of SMBs in monkeys. (B) Information about the incidence and gender of monkeys exhibiting SMBs. (C) Age distribution. (D) The SMB episodes of all monkeys for 14 consecutive days, illustrated as an example actogram of M2 under a normal 12-hour light/12-hour dark cycle. (E) (Top) Time distribution of the daily first and last SMB episode in monkeys with SMBs. (Bottom) The span between the first and last episodes. (F) The duration and frequency of daily SMB episodes in each monkey, presented as mean ± SEM. (G) The coefficient of variation (CV) of daily SMB episodes during the 14-day period. (H) The average single-episode duration of SMBs for all monkeys (*n* = 10), with the number in brackets following the monkey number in the legend representing the CV of the average single-episode duration of SMBs for that monkey. (I–K) The assessment of the long-term stability of SMB episodes in monkeys one year after the initial assessment, which includes the total daily duration (I), frequency (J) and average single-episode duration of SMBs (K). We obtained behavioral data (E–H) from monkeys M1–M8 on 14 consecutive days and monkeys M9 and M10 on 9 consecutive days. Statistical significance is indicated by *P < 0.05; **P < 0.01; ***P < 0.001; ns represents no significant difference.

To identify rhesus monkeys with spontaneous and persistent SMBs, we screened a total of 485 single-cage-reared rhesus monkeys from two monkey facilities (see Methods) and selected 31 candidates that exhibited repetitive motor behaviors. Among these candidates, we found 10 (5 males, 5 females) that met our criteria for persistent SMBs: performing two or more movements in a fixed sequence with a frequency greater than 100 times/day (Fig. 1A and B; Table 1). These criteria are analogous to compulsive sequential behaviors in humans with OCD [6]. Of the 10 monkeys, 8 were 3 to 4 years old (adolescence and early adulthood), and two others were 6 and 7 years old (Fig. 1C). We noted that all 10 monkeys identified were relatively young and had been single-cage reared for at least one month, during which SMBs appeared. Animal archives and medical records revealed no history of physical illness, drug therapy or prior experimentation.

**Table 1.** Phenomenology of sequential motor behaviors (SMBs) in monkeys.

**Subject**	**Gender**	**Age**	**SMBs (A, B, C…, actions in sequence)**
M1	Male	4	A: Rotating clockwise until the head faces the right rear corner of the cage; B: Rotating counterclockwise until the head faces the left front corner (the original position).
M2	Male	4	A: Standing up on two feet; B: Turning counterclockwise to the left rear of the cage; C: Crawling back to the original position.
M3	Female	4	A1: Flipping forward from the perch bar to the left side of the cage; B1: Standing on two feet and shaking on the left side. A2: Flipping forward from the perch bar to the left side of the cage; B2: Circling counterclockwise around the bottom of the cage.
M4	Female	4	A: Standing up on two feet and circling clockwise; B: Standing up on two feet and circling counterclockwise with forelimbs against the cage wall.
M5	Male	4	A: Standing up on two feet at the rear right of the cage with forelimbs against the cage wall; B: Bending over to the left to return to the crawling position; C: Circling counterclockwise once before returning to the original position.
M6	Male	4	A1: Crawling back and forth on the perch bar once; B1: Circling counterclockwise once and returning to the original position. A2: Crawling back and forth between the front lower-left corner and the back lower-right corner of the cage; B2: Circling counterclockwise.
M7	Female	3	A: Circling counterclockwise once; B: Standing up on two feet and then circling counterclockwise around the cage wall with the forelimbs against the cage wall.
M8	Female	3	A: Crawling back and forth once on the perch bar; B: Standing up on two feet on the bar and circling clockwise with the forelimbs against the cage wall.
M9	Female	7	A: Repeatedly circling the cage wall; B: Tossing the head; C: Repeatedly somersaulting.
M10	Male	6	A: Tossing the head; B: Circling counterclockwise; C: Repeatedly somersaulting.

Unique to the 10 monkeys (Table 1), the observed SMBs consisted of two or more elements that were either stereotypical movements (e.g. circling) or actions used in daily activities (e.g. standing up on the bar with two feet). Each element was performed in a precise sequence and location in the cage. Supplementary Movie 1 shows examples of SMBs for each monkey.

### Daily patterns and stability of SMBs

To assess SMBs in these monkeys, we collected behavioral data using video recordings for 14 consecutive days and measured the duration and frequency of SMB episodes, each consisting of uninterrupted motor sequences. Quantitative analysis of the SMBs, based on 1-min samples from each 10-min bin over the 24-hour cycle, showed that these monkeys spent a substantial amount of time (1.4 ± 1.1 h/day) engaging in SMBs, throughout their waking hours that began at 6 : 00–8 : 00 and ended at 19 : 00–20 : 00 (Fig. 1E). Furthermore, eight monkeys (all except M1 and M9) exhibited SMBs in the dark for a brief period immediately after lights out, as shown in the example M2 in Fig. 1D. Notably, six monkeys (M1, M2, M7, M8, M9 and M10) spent more than an hour per day engaged in SMBs (Fig. 1F).

All monkeys (except M6) showed low inter-individual variability in the total duration and frequency of daily SMB episodes during the 14-day period (coefficients of variation < 0.4; Fig. 1G). Furthermore, the average single-episode duration remained relatively constant for each monkey over time (Fig. 1H). The long-term stability of these SMBs in individual monkeys was further studied by re-examining the behavior of two monkeys one year after the initial assessment. We found that both monkeys exhibited similar sequential patterns, as well as total daily duration (Fig. 1I), frequency (Fig. 1J) and average single-episode duration (Fig. 1K) of SMBs during the reassessment. This long-term stability of SMBs points to the stability of the neural substrates underlying SMBs (see below).

### Identification of OCD risk gene mutations in monkeys with SMBs

**Figure 2.**
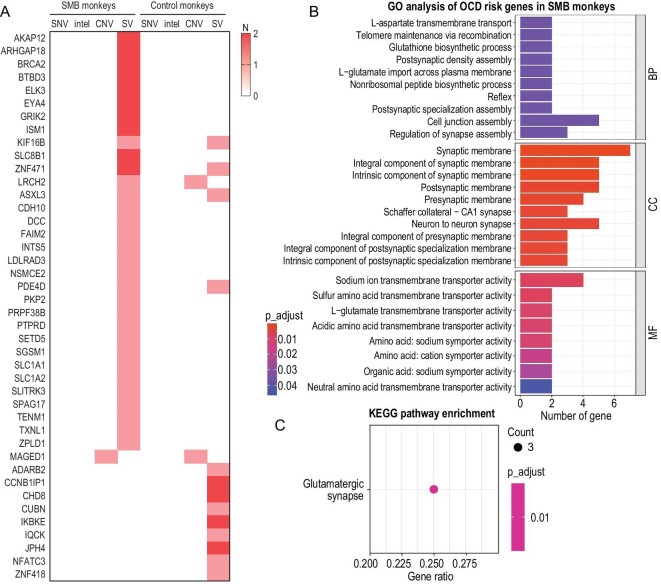
Genetic analysis conducted on the risk genes associated with OCD in monkeys with SMBs. (A) Four types of gene variations present in both groups, including single-nucleotide variations (SNVs), insertions-deletions (indels), copy number variants (CNVs) and structural variants (SVs). (B and C) Enrichment analyses of the OCD risk genes in the SMB monkeys using Gene Ontology (GO) (B) and the Kyoto Encyclopedia of Genes and Genomes (KEGG) (C). GO and KEGG analysis were performed using the clusterProfiler package (version 4.6.2) within R software.

The infrequent occurrence of persistent SMBs in single-caged monkeys suggests that genetic factors may contribute to their emergence, in a way that is possibly similar to the role of genetics in the development of OCD in humans [16–19]. DNA samples from 24 unrelated monkeys, including 8 with SMBs and 16 control monkeys without SMBs, were subjected to whole-genome sequencing. These variants included 501 single nucleotide variants (SNVs), 912 insertions and deletions (indels), 804 copy number variants (CNVs) and 12 931 structural variants (SVs) that were identified as severely damaging singleton mutations (see Methods), and were mapped to 10 165 genes. We compiled a list of 109 risk genes implicated in human OCD (Table S1) to identify potential gene mutations associated with OCD risk in monkeys. Mutations on these candidate risk genes were extracted for analysis. A comparison of the distribution of severely damaging variants in OCD risk genes versus other genes in SMB monkeys and controls showed a higher ratio of severely damaging variants in OCD risk genes to severely damaging variants in other genes in SMB monkeys compared to controls (odds ratio (OR) = 1.60, *P* = 0.031). Specifically, SMB monkeys had 44 severely damaging variants in OCD risk genes, whereas only 19 severely damaging variants in OCD risk genes were detected in controls. Among these variants, 95% were SVs and 5% were CNVs (Fig. 2A). In addition, there was no significant difference in the ratio of severely damaging variants to benign variants in SMB monkeys compared to controls (OR = 1.05, *P* = 0.284). We then performed Gene Ontology and Kyoto Encyclopedia of Genes and Genomes enrichment analysis to explore the potential pathways affected by these mutations and found that they were associated with the synaptic connection dysfunction (Fig. 2B and C; adjusted *P* < 0.05), which may be related to alterations in cortico–striatal–thalamic–cortical (CSTC) circuitry. We noted that genetic risk factors such as *AKAP12* (detected twice), *GRIK2* (detected twice), *KIF16B* (detected twice) and *PTPRD* were identified exclusively in monkeys with SMBs. These mutations may have a role in the development of SMB in monkeys. Additionally, this monkey–human cross-validation may help to delineate genetic factors associated with OCD in humans.

### Alterations of brain structures in monkeys with SMBs

**Figure 3.**
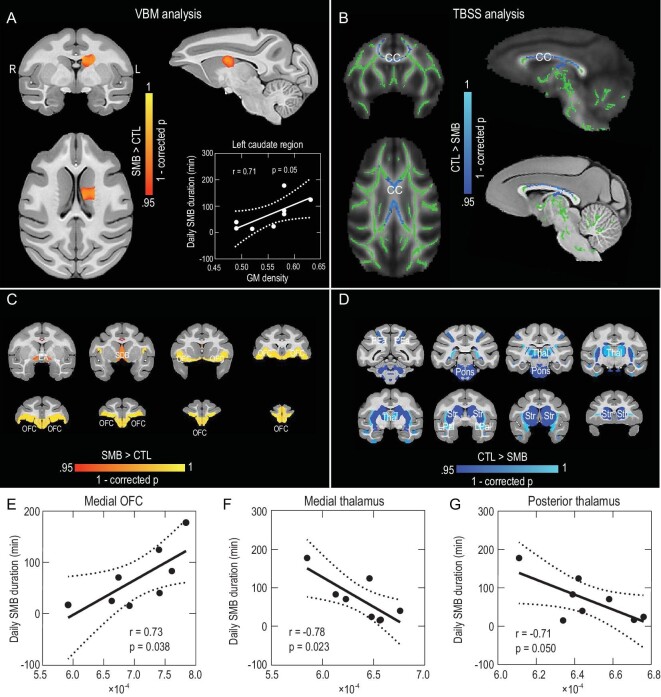
Alterations of brain structures in monkeys with SMBs. (A) Voxel-based morphometry analyses show larger GM volumes in the left caudate nucleus of SMB monkeys. Red-yellow indicates monkeys with SMBs greater than controls; the color bar denotes a 1-corrected *P*-value range of 0.95–1.0. Bottom right panel: correlation between the daily SMB duration and regional GM volumes in the left caudate nucleus. (B) Tract-based spatial statistics analyses show that SMB monkeys had significantly lower FA values than controls, as represented by the dark/light blue color scale. The color bar denotes a 1-corrected *P*-value range of 0.95–1.0. (C and D) Regions in which there was a significant change in DTI parameter MD. Red-yellow represents regions where SMB monkeys had higher MD values than controls (C), whereas dark/light blue represents regions where controls had higher MD values than SMB monkeys (D). The color bar shows a 1-corrected *P*-value range of 0.95–1.0. (E–G) Correlation between the daily SMB duration and MD values in the medial OFC (E), medial thalamus (F) and posterior thalamus (G). Abbreviations: VBM, voxel-based morphometry; GM, gray matter; TBSS, tract-based spatial statistics; DTI, diffusion tensor imaging; MD, mean diffusivity; FA, fractional anisotropy; CC, corpus callosum; EA, extended amygdala; SDB, septum diagonal band; OFC, orbital frontal cortex; PEa, area PEa, Brodmann's Area 5; Thal, thalamus; LPal, lateral pallium; Str, striatum; CTL, control.

To investigate the neural basis of SMBs in these monkeys, we evaluated the differences in the gray and white matter structures between eight monkeys with SMBs and eight normal monkeys without SMBs, using MRI with T1-weighted (T1w) imaging and diffusion tensor imaging (DTI), respectively. We first analyzed the T1w imaging data using voxel-based morphometry (VBM), involving a voxel-wise comparison of the local gray matter (GM) volume between groups of subjects [20,21]. These VBM analyses showed that the only significant difference was a larger average GM volume in the left caudate nucleus of monkeys with SMBs compared to that of control monkeys (Fig. 3A and S1A). Furthermore, this GM volume positively correlated with the duration of daily SMB episodes (Fig. 3A; r = 0.71, *P* = 0.05), although such correlation was not found when using a voxel-wise evaluation.

We next analyzed the DTI data to evaluate white matter (WM) properties of monkeys with SMBs using tract-based spatial statistics (TBSS), a voxel-wise analysis of multi-subject diffusion data [22]. The TBSS analysis revealed that the fractional anisotropy (FA) value of the corpus callosum (CC) in monkeys with SMBs was lower than that found in control monkeys (Fig. 3B and S1B), indicating compromised WM integrity in this region [23]. No differences in FA values were found in other brain areas. This TBSS result is consistent with the finding of a lower FA in the CC of adult OCD patients from human imaging studies [24–30].

We also compared the mean diffusivity (MD, a DTI parameter) between the two monkey groups in brain regions defined by the Cortical Hierarchy Atlas (CHARM [31,32]) and the Subcortical Atlas (SARM [33]) of the Rhesus Macaque. Compared to the control group, SMB monkeys had altered MD throughout the brain, with 61.5% of abnormalities found in components of the CSTC circuitry. These abnormalities mainly included higher MD values in the orbital frontal cortex (OFC) and lower MD values in the striatum and thalamus (Fig. 3C and D; Table S2; *t*-test, *P* < 0.05). Further correlation analysis of these abnormal brain regions revealed significant associations with the duration of daily SMB episodes. Specifically, MD values in the medial OFC showed a positive correlation with the duration of daily SMB episodes (Fig. 3E; r = 0.73, *P* = 0.038). Moreover, the MD values in both the medial and posterior thalamus showed a negative correlation with the duration of daily SMB episodes (Fig. 3F and G; medial: r = -0.78, *P* = 0.023; posterior: r = -0.71, *P* = 0.050). Thus, there were significant microstructural changes in the CSTC circuitry of SMB monkeys. Taken together, the structural changes in both CC and CSTC circuitry may be related to the development and maintenance of SMBs.

### The effect of external stimuli on SMBs in monkeys

**Figure 4.**
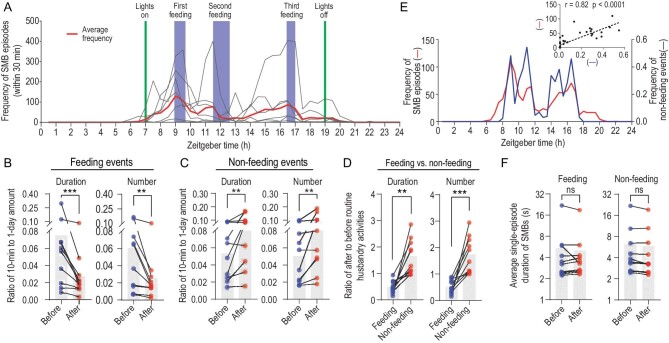
External stimulus-evoked SMB episodes in monkeys. (A) The distribution pattern of SMB episode frequency over a 24-hour period (*n* = 8). Frequency is defined as the total number of SMB episodes during a 30-min interval. (B) The effect of feeding events on SMB duration and frequency during the 10-min period before and after the events, including first, second and third feeding (*n* = 10). (C) The effect of non-feeding events on SMB duration and frequency during the 10-min period before and after the events (*n* = 10). (D) Comparison of the effect of feeding events and non-feeding events on SMB duration and frequency. (E) The 24-hour distribution of the average SMB frequency and non-feeding events (number of events per 30-min interval). The inset shows a positive correlation between SMB frequency and non-feeding-event frequency, with data pooled from 10 monkeys. (F) The average single-episode duration of SMBs during the 10-min period (*n* = 10) before and after feeding events and non-feeding events. Statistical significance is indicated by *P < 0.05; **P < 0.01; ***P < 0.001; n.s., not significant. Two-tailed paired *t*-tests were used in all panels.

A range of external stimuli may evoke symptoms in individuals with OCD. To determine the stimulus-evoked SMB episodes in monkeys reared under standard cage conditions, we analyzed the correlation between SMB occurrences and routine husbandry activities, including feeding and non-feeding events such as cleaning and animal inspection (i.e. human presence without feeding, see Methods). We found that SMB frequency showed progressive elevation before feeding and a rapid drop afterwards (Fig. 4A and S2A–B). Further quantification of the effect of feeding showed that the duration and number of SMB episodes during the 10-min period after the feeding events were 49 ± 23% and 51 ± 25% of those during the 10-min period before the events, respectively (Fig. 4B; paired *t*-test, *n* = 10; *P* < 0.001, duration; *P* = 0.002, number). In contrast, the duration and number of SMB episodes during the 10-min period after non-feeding events were 165 ± 67% and 174 ± 69% of those observed prior to the events, respectively (Fig. 4C; paired *t*-test, *n* = 10; *P* = 0.008, duration; *P* = 0.004, number). These results suggest a negative association between SMBs and the monkeys’ state of hunger, and a positive association with the presence of humans. Furthermore, the effects of non-feeding events were significantly higher than those of feeding events (Fig. 4D; paired *t*-test, *n* = 10; *P* = 0.001, duration; *P* < 0.001, number). We also found a positive correlation between SMB frequency and non-feeding frequency during daytime (Fig. 4E; Pearson's r = 0.82, *P* < 0.0001). In addition, we found that the average single-episode duration of SMBs was relatively constant before and after both types of events (Fig. 4F; paired *t*-test, *n* = 10; *P* = 0.310, feeding events; *P* = 0.177, non-feeding events). Taken together, these results suggest that SMBs are not random behaviors but reflect the monkeys’ internal states and external stimuli.

### Fluoxetine partially alleviates SMBs in monkeys

**Figure 5.**
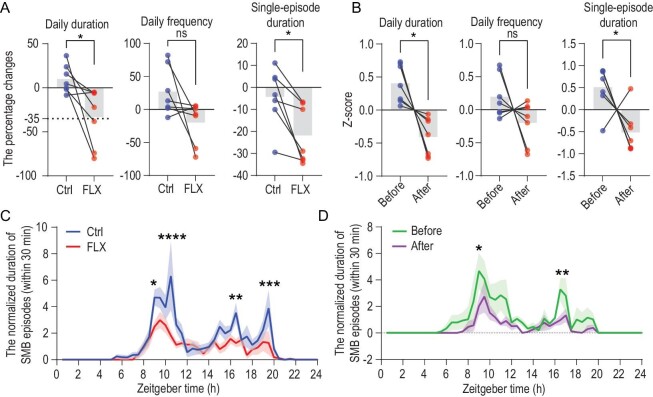
The effect of fluoxetine treatment on SMB episodes in monkeys. (A) Comparison of the percentage changes in the total daily duration, frequency and average single-episode duration of SMBs in monkeys treated with fluoxetine for 8 weeks, in comparison with those treated with vehicle (crossover design, *n* = 7). (B) Z-scores of the same parameters before and after treatment in fluoxetine-treated monkeys (*n* = 7), with the raw data transformed using z-scores. (C) Comparison of the 24-hour distribution of SMB duration between fluoxetine-treated and vehicle-treated monkeys. SMB duration, measured in 30-min bins, was divided by the duration at baseline for data normalization. (D) Comparison of the 24-hour distribution of SMB duration before and after fluoxetine treatment for responders, referring to monkeys who exhibited at least 35% reduction in daily SMB duration following fluoxetine treatment. Abbreviations: Ctrl, control; FLX, fluoxetine. Statistical significance is indicated by *P < 0.05; **P < 0.01; ***P < 0.001; ns represents no significant difference.

Selective serotonin reuptake inhibitors (SSRIs), such as fluoxetine, are the first line pharmacotherapy for OCD [34]. We evaluated the effect of fluoxetine on monkeys with SMBs by comparing the percentage changes in total daily duration, average frequency and average single-episode duration of SMBs after 8 weeks of fluoxetine treatment with those after vehicle treatment. Group comparison of the percentage changes due to the treatment showed differences in total daily duration, average single-episode duration and average frequency of SMBs with total daily duration and average single-episode duration reaching statistical significance (Fig. 5A; paired *t-*test, *n* = 7, crossover design; *P* = 0.030, total duration; *P* = 0.094, frequency; *P* = 0.011, single-episode duration). Fluoxetine treatment significantly reduced the daily duration (33 ± 33%; paired *t-*test, *n* = 7, *P* = 0.010) and single episode duration (22 ± 13%; paired *t-*test, *n* = 7, *P* = 0.033), whereas vehicle treatment had no significant effect on these parameters (Fig. 5B and S3A). Further analysis of the daily pattern of SMBs after fluoxetine treatment revealed that the reduction in SMBs occurred primarily during high-frequency periods of the behavior, with no significant change during low-frequency periods (Fig. 5C). Furthermore, three out of the seven monkeys displayed a reduction of at least 35% in the daily duration of their SMBs, primarily during the high-frequency periods of the behavior (Fig. 5D). These results are consistent with patterns of treatment response after fluoxetine in humans with OCD, and further support the notion that the SMBs in these monkeys are a valid experimental model of obsessive-compulsive behavior.

## DISCUSSION

OCD affects 2%–3% of the global population and is known to cause significant distress and disability, leading to a substantial economic burden for individuals, families and societies [2,35]. In the present study, we have identified a group of single-caged rhesus monkeys exhibiting spontaneous SMBs. These SMBs closely resemble human OCD rituals and allow for quantitative analysis. Remarkably, we have found that these monkeys carry severely damaging variants in genes associated with OCD risk in humans, show alterations in neurocircuitry that are congruent with those seen in neuroimaging studies of OCD, and demonstrate similar patterns of response to SSRIs as are seen when these agents are given to humans with OCD. The presence of SMBs in monkeys provides a valuable experimental model for investigating the neurobiology of OCD. These findings build on earlier literature on rodent stereotypies as an experimental model for OCD [36], extending this in novel directions with the more fine-grained genetic and neuroimaging analyses that an NHP species allows.

Persistent SMBs are present in ∼2% of single-caged monkeys, consistent with prevalence estimates from epidemiological studies of OCD in humans. Furthermore, the features of monkey SMBs observed in this study are reminiscent of the characteristics of ritualistic behaviors in humans with OCD in that both behaviors were repetitive, time-consuming and stable over prolonged periods. Finally, our findings suggest a multifactorial origin that involves both genetic and environmental factors, in line with our understanding of the pathogenesis of human OCD [16–19].

SMBs emerged soon after the monkeys were moved to single cages, and were exacerbated by a particular stressor (human presence). Long-term isolation in the single-cage rearing condition is associated with the development of SMBs in macaques [37], and studies of captive animals (such as bears [6]) suggest that SMBs can be induced by confinement in a relatively small space. Analogously, the literature on OCD in humans indicates that exposure to environmental stressors is an important risk factor for the development and exacerbation of this condition [38–40].

Genetic risk factors appear to be important contributors to the development of SMBs in monkeys. Remarkably, monkeys with SMB were significantly more likely to have severely damaging mutations in genes that are thought to play a role in OCD. Although SVs were the most prominent mutations found in OCD risk genes in monkeys with SMBs, the number of severely damaging SVs in each monkey did not correlate with symptom severity. Analogously, the literature on OCD in humans indicates a complex genetic architecture, with rare variants playing some role, and with a range of common variants also contributing to risk [17,41]. The finding that some individuals in the control group have severely damaging mutations is also consistent with the human literature [42], and underscores the complexity of the mechanisms that underpin SMBs; a range of gene variants and environmental factors may interact to determine risk for and resilience against these behaviors. While large samples are needed to conduct a comprehensive genome-wide association analysis, in smaller samples an approach that focuses on severe damaging genes may be useful [42].

The basal ganglia have long been considered mediators of OCD symptoms [43], and many neuroimaging studies have shown OCD-associated increases in the GM volume and metabolism/activation of the striatum [29,44–49]. Consistent with human findings [45,50], we found that SMB monkeys had larger caudate nuclei than control monkeys. Notably, caudate nucleus GM volume correlated positively with daily duration of monkey SMBs. Similarly, SMB monkeys had low FA values in the CC, consistent with compromised WM integrity, and congruent with findings in human OCD [24–30]. SMB monkeys had MD values in many CSTC regions (including the OFC and thalamus [51]) that were significantly different from those of control monkeys, suggesting structural changes in regions besides the caudate nucleus and CC. This is consistent with the OCD literature that suggests that multiple regions of the brain play a role. The neural mechanism by which external stressors affect monkey SMBs as well as human OCD remains to be fully understood, and may be a fruitful avenue for further investigation.

Fluoxetine decreased SMBs in our monkeys, with 42.9% of monkeys demonstrating a marked effect. This is consistent with the patterns of treatment responses seen in humans with OCD and suggests that the serotonin system may play a role in the neurobiology of SMBs in monkeys. This is consistent with a range of other evidence suggesting that the serotonin system contributes to stereotypic behaviors in animals [52,53] and in humans [54]. Future work on the response of SMB in monkeys to the range of medications that have proven efficacious for OCD in humans may be useful in further validating the SMB experimental model.

The widespread use of spontaneous animal models of human disease may be limited by the difficulty in identifying the disease-related phenotype and the number of animals identified. While monkeys with SMBs were not difficult to identify, their numbers were small. With rapid advances in reproductive technologies for NHPs, including assisted reproduction [55,56], cloning by somatic cell nuclear transfer [57,58] and accelerated reproduction [59,60], it is possible to help generate adequate numbers of SMB monkeys with more uniform genetic backgrounds and fixed SMB phenotypes. This would allow additional work aimed at consolidating and extending this experimental model of OCD, with the ultimate aim of developing new OCD therapies.

## MATERIALS AND METHODS

For details about materials and methods, please see the Supplementary Data.

## Supplementary Material

nwad312_Supplemental_Files
