# Supplementary material for: Rhesus monkeys exhibiting spontaneous ritualistic behaviors resembling obsessive-compulsive disorder
Source: Natl Sci Rev. 2023 Dec 8;10(11):nwad312. doi: 10.1093/nsr/nwad312 (PMC10751879; doi:10.1093/nsr/nwad312)
Supplement: nwad312_Supplemental_Files [file nwad312_supplemental_files.zip › Zhai.Supplemental file.docx]

Supplementary data

**Rhesus monkeys exhibiting spontaneous ritualistic behaviors resembling obsessive-compulsive disorder**

Rongwei Zhai^1,2,3,4,†^*, Geya Tong^1,†^, Zheqin Li^1^, Weichen Song^1^, Yang Hu^1^, Sha Xu^2,3^, Qiqi Wei^2,3^, Xiaocheng Zhang^2,3^, Yi Li^1^, Bingbing Liao^1^, Chenyu Yuan^1^, Yinqing Fan^1^, Ge Song^1^, Yinyin Ouyang^1^, Wenxuan Zhang^1^, Yaqiu Tang^1^, Minghui Jin^1^, Yuxian Zhang^2^, He Li^2^, Zhi Yang^1^, Guan Ning Lin^1^, Dan J. Stein^5^, Zhi-Qi Xiong^2,4^*, Zhen Wang^1,6^*

^1^Shanghai Mental Health Center, Shanghai Jiao Tong University School of Medicine, Shanghai, China.

^2^Institute of Neuroscience, State Key Laboratory of Neuroscience, CAS Center for Excellence in Brain Science and Intelligence Technology, Chinese Academy of Sciences, Shanghai 200031, China.

^3^Lingang Laboratory, Shanghai 200031, China

^4^Shanghai Center for Brain Science and Brain-inspired Technology, Shanghai 201602, China

^5^South African Medical Research Council on Risk & Resilience in Mental Disorders, Department of Psychiatry & Neuroscience Institute, University of Cape Town, South Africa.

^6^Shanghai Key Laboratory of Psychotic disorders, Shanghai Mental Health Center, Shanghai Jiao Tong University School of Medicine, Shanghai, China.

*Corresponding authors: Zhen Wang [(wangzhen@smhc.org.cn);](mailto:(wangzhen@smhc.org.cn);) Zhi-Qi Xiong ([xiongzhiqi@ion.ac.cn](mailto:qsun@ion.ac.cn)); Rongwei Zhai (zhairw@lglab.ac.cn)

†Equally contributed to this work.

Methods

**Animal Ethics Statement**

The use and care of rhesus monkeys (*Macaca mulatta*) in this study complied with the guidelines of the Animal Advisory Committee at the Shanghai Institute of Biological Science, Chinese Academy of Sciences/Center for Excellence in Brain Science and Intelligence Technology, Chinese Academy of Sciences. The ethics application titled “Exploration of intervention mechanisms and novel targets using non-human primate model of mental disorders” (#ER-SIBS-221508P) was approved by the Shanghai Institute of Biological Science, Chinese Academy of Sciences.

In this study, to identify rhesus monkeys with spontaneous sequential motor behaviors (SMBs), we screened a total of 485 single cage-reared rhesus monkeys from two monkey facilities (see below) and selected 31 candidates that exhibited repetitive, time-consuming (> 0.25 hours/day), and stable (coefficient of variation < 0.6 over 2 weeks) motor behaviors. Among these 31 candidates, we then identified 10 rhesus monkeys aged 3 and 7 years, of which 5 were male and 5 were female, that exhibited SMBs. These SMBs were characterized by two or more sequence elements, including stereotyped movements such as circling and somersaulting, as well as daily activities like standing on the bar with two feet. All of the monkeys were in good health and were selected exclusively for this study. Six of them (3 males and 3 females) came from a group of 240 individually reared monkeys at Tiangen Biotechnology Institute in Hubei, China. The other four (2 males and 2 females) came from a group of 245 individually reared monkeys at the Suzhou Non-Human Primate Facility of the Center for Excellence in Brain Science and Intelligence Technology (Institute of Neuroscience).

The monkeys were housed individually in a temperature-controlled environment (temperature: 22 ± 1℃) with 50% ± 5% humidity and a 12-hour light/dark cycle (lights on from 07:00 to 19:00). They were fed twice daily with commercial monkey chow (Anmufei, Suzhou) fruits once daily, and had free access to water. Toys were also provided, and the monkeys were carefully observed by veterinarians to ensure their health during and after the experiments.

**Behavioral Assessment**

Monkeys’ behavior was recorded throughout the day using a high-resolution video recording system with six infrared cameras (maximum resolution 1920 x 1080, frame rate 25 Hz) and three hard disk recorders (Hangzhou Hikvision Digital Technology, Zhejiang, China), covering the entire monkey cage. Behavioral observations were conducted on all 10 monkeys in their living cages under normal living conditions. The duration and number (count) of SMBs were quantified during the first minute of each 10-minute interval over 24 hours of each day, spanning 14 consecutive days for monkeys M1-M8 and 9 consecutive days for monkeys M9-M10. If no data was available for a minute (e.g., the monkey’s scene was obscured), it was replaced by the next one. If no data was available for 10 minutes, the behavioral value for that time period was recorded as missing and replaced by the average of its two nearest neighbors. Daily episode duration and frequency were obtained by multiplying daily sample’s total duration and frequencies by ten, respectively. The ratio of total daily duration to frequency equaled the average single-episode duration of SMBs. Trained observers blinded to experimental conditions recorded all SMBs (Table 1), with inter-observer reliability exceeding 85%.

**Stability of SMBs**

To assess the stability of SMBs in monkeys, we calculated the coefficient of variation (CV) for the duration and frequency of SMB episodes for each monkey over 14-day periods. For long-term stability, we conducted a follow-up study on two untreated monkeys a year after the initial assessment, measuring their SMBs for 14 consecutive days similarly to the method described in the ‘Behavioral assessment’ section.

**Genetic Analysis**

Genomic DNA was extracted from whole-blood samples collected from the 24 rhesus monkeys (the cohort was comprised entirely of unrelated monkeys, as detailed in Table S3) and sent to BGI-Shenzhen (Shenzhen, China) for processing. DNA extraction and library preparation followed the standard BGI protocols. Briefly, red blood cells were lysed, and DNA was extracted using phenol-chloroform-isoamyl alcohol extraction. The extracted DNA was assessed using Qubit fluorometer and gel electrophoresis to ensure quality. Whole-genome sequencing libraries were constructed from fragmented genomic DNA ranging from 200 to 400 bp, which were subjected to end-repair, 3' adenylation, adapter ligation, and PCR amplification. The qualified libraries were then sequenced on the BGISEQ-500 platform.

The raw sequencing data underwent processing to eliminate low-quality reads and unidentified bases. Data cleanliness was then assessed using FastQC (version 0.11.5). Clean data were aligned to the rhesus monkey reference genome (https://ftp.ncbi.nlm.nih.gov/genomes/all/GCF/003/339/765/GCF_003339765.1_Mmul_10/GCF_003339765.1_Mmul_10_genomic.fna.gz; the reference genome version is GCF_003339765.1_Mmul_10) using BWA (version 0.7.17). Picard (version 2.18.26) was used to remove duplicate sequence reads. Further realignment was performed using the Genome Analysis Toolkit (GATK, version 4.2.6.1). Single-nucleotide variations (SNVs) and insertions-deletions (indels) were called using HaplotypeCaller of GATK and annotated with SnpEff software (version 4.1). Copy Number Variants (CNVs) were called using the CNVnator read-depth algorithm, while Manta (version 1.6.0) was used to identify structural variants (SVs) with standard settings.

Four types of variations, including SNV, indel, CNV, and SV, were identified, excluding recurrent mutations. Frameshift and stop gain/loss variations for SNV and indel, and CNVs and SVs overlapping with coding regions and untranslated regions, were classified as severely damaging mutations. Other variations were classified as benign. The study quantified the number of severely damaging mutations and benign variants for each gene for the 24 monkeys. We compiled a list of genes linked with human OCD (Table S1). This list included genes with common variants associated with OCD that were reported in two genome-wide association studies [1, 2] as well as genes with rare de novo mutations found from whole-genome sequencing of parent-offspring OCD trios [3]. The distribution of severely damaging variants in OCD risk genes versus other genes in SMB monkeys and controls was then compared using the Fisher test, and an odds ratio (OR) calculated. In addition, the distribution of severely damaging variants versus benign variants in SMB monkeys and controls was compared, again using the Fisher test, along with the calculation of an OR. Gene Ontology (GO) enrichment and Kyoto Encyclopedia of Genes and Genomes (KEGG) pathway analysis of target gene sets were performed using the clusterProfiler package [4, 5] (version 4.6.2) within R software.

**MRI Acquisition**

A cohort of 16 rhesus monkeys, comprising 8 with SMBs and 8 without, underwent whole-brain T1-weighted (T1w) and diffusion-weighted (DW) scans using a Siemens Tim Trio 3T scanner (Erlangen, Germany) at the Center for Excellence in Brain Science and Intelligence Technology (Institute of Neuroscience), Chinese Academy of Sciences. On the day of the scan, subjects were subjected to food restriction prior to receiving intramuscular injections of either ketamine (10 mg/kg) or Zoletil 50 (50 mg/ml, 5 mg/kg) anesthesia, and atropine sulfate (0.05 mg/kg). Additional local anesthetic (5% lidocaine cream) was applied around the ears to block peripheral nerve stimulation. Anesthetized animals were then placed in a custom-built MRI-compatible stereotaxic frame in the sphinx position. During the scan, the animals were maintained in the anesthetic state using the lowest possible isoflurane concentration (ranging from 0.8-1.5%). High-resolution T1w images were obtained using an MPRAGE sequence with the following parameters: time repetition (TR) = 2300; time echo (TE) = 3 ms; inversion time = 1000 ms; flip angle (FA) = 9°; matrix size = 192 x 192; field of view (FOV) = 96 mm x 96 mm; slice thickness = 0.5 mm; acquisition voxel size = 0.5 x 0.5 x 0.5 mm^3^. Five to seven T1w MR volumes were acquired and averaged to achieve high signal-to-noise ratio (SNR). Each Diffusion-weighted image (DWI) scan was acquired using a 2D echo-planar imaging sequence with the following parameters: TR = 9000 ms; TE = 81 ms; FA = 90°_;_ FOV = 83 mm x 83 mm; matrix size = 64 x 64; slice thickness = 1.3 mm, no gaps, acquisition voxel size = 1.3 x 1.3 x 1.3 mm^3^, b = 1000 s/mm^2^, 64 directions with 10 b0 images, with diffusion data acquired in both A-P and P-A phase encoding directions for subsequent DWI distortion correction, each scanned five times.

**Image Analyses**

This multimodal neuroimaging study employed two neuroimaging methods: voxel-based morphometry (VBM) analysis on gray matter (GM) volume and tract-based spatial statistics (TBSS) on white matter (WM) microstructure. Image analysis was performed using tools from the FMRIB Software Library (FSL) software (Oxford Centre for Functional Magnetic Resonance Imaging of the Brain Software Library, <http://www.fmrib.ox.ac.uk/fsl>) [6], with a brief description of the preprocessing and processing steps as follows.

**Voxel-Wise Morphometry**

To preprocess and analyze the images, T1w images were first rigidly aligned to the first and then averaged within-subject using the linear image registration tool FLIRT in FSL. The FSL-BET automatic tool [7] and manual skull-stripping were used for brain extraction of the within-subject average. Following bias field correction, the preprocessed T1w images were then divided into three tissue compartments, including GM, WM, and cerebrospinal fluid, using FSL-FAST [8, 9]. After segmenting these compartments, FSL-VBM [10, 11] was utilized for analysis The segmented GM partial volume images were aligned to the GM volume of NIMH Macaque Template (NMT) [12] using FLIRT [6, 13] and the nonlinear registration tool FNIRT [14] in FSL. The resulting images were averaged to create a study-specific template, to which all native GM images were nonlinearly re-registered. The registered partial GM volume images were modulated by multiplying by the Jacobian determinant of the warp field to correct for local expansion or contraction due to the nonlinear component of the transformation. The modulated segmented images were smoothed with an isotropic Gaussian kernel with a sigma of 1.5 mm. Finally, voxel-wise general linear modeling (GLM) was applied using permutation-based nonparametric tests and threshold-free cluster enhancement (TFCE), with multiple comparison correction across space using familywise error rate (FWE) correction.

**Diffusion Tensor Imaging**

Voxel-wise statistical analysis of fractional anisotropy (FA) data was performed using TBSS [15] in FSL. Briefly, DWIs were corrected for eddy currents and head motion using FMRIB’s Diffusion Toolbox (FDT) [16], part of FSL. Diffusion data were collected with reversed phase-encode blips, resulting in pairs of images with distortions going in opposite directions. The susceptibility-induced off-resonance field was estimated from these image pairs using the Topup tool [17] implemented in FSL, with each paired image combined into one corrected image. These DWIs were rigidly aligned to the first using FLIRT and then averaged within-subject to improve the SNR. High-quality b0 masks were obtained by transforming T1w masks from T1w space to native DWI space using a linear transformation matrix between T1w and DWI. The FDT was then used to calculate diffusion tensor-based measurements. To determine the most representative FA image as the target image, we estimated the average amount of warping required to align all other FA images to each FA image. The target image had the smallest amount of average warpings when used as a target. This target image was then linearly aligned to a monkey DTI template [18] to create a study-specific target, to which all FA images were nonlinearly re-registered. The mean FA image was created and thinned to form a mean FA skeleton representing the centers of all tracts common to the group. All aligned FA data were projected onto this mean FA skeleton and fed into voxel-wise cross-subject statistics. Finally, voxel-wise GLM was applied using permutation-based nonparametric tests and TFCE with multiple comparison correction across space using the FWE correction.

We compared mean diffusivity (MD) values between the two groups for all brain regions defined by the Cortical Hierarchy Atlas (CHARM [19, 20]) and Subcortical Atlas (SARM [21]) of the Rhesus Macaque, which offer a standardized parcellation for ROI and network analyses. Both atlases are in the space of the updated NIMH Macaque Template (NMT v2), a high-resolution population template based on *in vivo* scans collected at high field strength (4.7 T) from a large cohort (N = 31) of adult rhesus monkeys [12, 20]. Results were corrected for multiple comparisons using false discovery rate (FDR) (p < 0.05).

**Behavior-imaging Correlations**

We examined the correlation between symptom severity, measured by daily SMB duration, and GM volume using a linear regression model. This model tested whether GM volume (voxel-wise) would predict daily SMB duration with age and gender as covariates and correcting for multiple comparisons using FWE after applying TFCE (p < 0.05). We then used a linear regression model to examine the relationship between daily SMB duration and the GM volume and MD values of each significant region identified in the VBM analysis and DTI measurements, respectively.

**Effect of Husbandry Activities on SMBs**

To assess the effects of routine husbandry activities on monkey SMBs, we categorized these routines into two groups: feeding events (i.e., first, second, and third feeding) and non-feeding events (i.e., room cleaning, cage cleaning, and animal inspection). We measured the duration and number of SMBs during a 10-minute period before and after each event over 14 or 9 days (refer to ‘Behavioral assessment’ section), then analyzed the association between SMB occurrences and these events. For feeding events, we used event markers to indicate event-related SMB episodes throughout the day. We divided 24 hours into 48 30-minute bins and calculated the cumulative frequency of SMB episodes during each bin. For non-feeding events, we performed Pearson correlation analysis to examine the relationship between SMB episode frequency and event frequency. We also examined the impacts of these events on the duration and frequency of SMB episodes by calculating the ratio of post-event to pre-event episodes.

**Fluoxetine Treatment**

To assess the efficacy of fluoxetine, a selective serotonin reuptake inhibitor (SSRI), which are first-line medications for OCD treatment, on SMBs in monkeys, we utilized a crossover design. Each monkey was given different treatments (fluoxetine or vehicle) at different time periods, with a crossover from one treatment to the other during assessment. Fluoxetine doses were determined based on preliminary pharmacokinetics [22] and previous studies in rhesus monkeys [23-30]. Fluoxetine, which generally takes up to eight weeks to take effect, was given to seven monkeys for eight weeks in random order, with a washout period of at least six months between fluoxetine and vehicle treatments to avoid carryover effects. The initial dose of fluoxetine was 2 mg/kg/day for the first week, followed by a constant dose of 4 mg/kg/day for the test treatment period. At the end of the treatment, a two-day half-dose tapering period was implemented. Fluoxetine, dissolved in 50 ml of drinking water, was administered orally daily at the same time (9:30 - 10:00), and all animals took 100% of their daily medication and were allowed to drink freely after administration.

SMB episodes were evaluated individually in each monkey at baseline and after eight weeks of treatment by raters blinded to which animals received which intervention. We used the percentage formula to estimate the change in SMB frequency and duration after treatment. This formula obtains the change rate by subtracting the initial value from the final value, dividing the result by the initial measurement value, and then multiplying it by 100.

**Statistical Analysis**

The behavioral data were analyzed using ANOVA or paired *t*-tests, as prescribed in the GraphPad Prism version 9.0.0 for Windows (GraphPad Software, San Diego, CA, USA). Paired or unpaired *t*-tests were used to compare SMB episodes before and after feeding or non-feeding events. For MD data, *t*-tests were used to compare the differences in ROI between the two groups with FDR correction applied to adjust for multiple comparisons. In analyzing the fluoxetine treatment data, we employed paired *t*-tests to compare the percentage changes in SMBs between the two groups, as well as z-scores of SMBs before and after treatment. Additionally, a two-way ANOVA was applied to compare the 24-hour distribution of SMB episodes between two groups. We also used the ANOVA to compare SMB episodes before and after fluoxetine treatment, with Šidák corrections for multiple comparisons.

**References**

1. Stewart, SE, Yu, D, Scharf, JM*, et al.* Genome-wide association study of obsessive-compulsive disorder. *Mol Psychiatry*. 2013; **18**(7): 788-98.

2. Mattheisen, M, Samuels, JF, Wang, Y*, et al.* Genome-wide association study in obsessive-compulsive disorder: results from the OCGAS. *Mol Psychiatry*. 2015; **20**(3): 337-44.

3. Lin, GN, Song, W, Wang, W*, et al.* De novo mutations identified by whole-genome sequencing implicate chromatin modifications in obsessive-compulsive disorder. *Sci Adv*. 2022; **8**(2): eabi6180.

4. Yu, G, Wang, LG, Han, Y*, et al.* clusterProfiler: an R package for comparing biological themes among gene clusters. *Omics : a journal of integrative biology*. 2012; **16**(5): 284-7.

5. Wu, T, Hu, E, Xu, S*, et al.* clusterProfiler 4.0: A universal enrichment tool for interpreting omics data. *Innovation (Cambridge (Mass))*. 2021; **2**(3): 100141.

6. Jenkinson, M, Bannister, P, Brady, M*, et al.* Improved optimization for the robust and accurate linear registration and motion correction of brain images. *Neuroimage*. 2002; **17**(2): 825-41.

7. Smith, SM. Fast robust automated brain extraction. *Human brain mapping*. 2002; **17**(3): 143-55.

8. Zhang, Y, Brady, M, Smith, S. Segmentation of brain MR images through a hidden Markov random field model and the expectation-maximization algorithm. *IEEE transactions on medical imaging*. 2001; **20**(1): 45-57.

9. Smith, SM, Jenkinson, M, Woolrich, MW*, et al.* Advances in functional and structural MR image analysis and implementation as FSL. *Neuroimage*. 2004; **23 Suppl 1**: S208-19.

10. Ashburner, J, Friston, KJ. Voxel-based morphometry--the methods. *Neuroimage*. 2000; **11**(6 Pt 1): 805-21.

11. Good, CD, Johnsrude, IS, Ashburner, J*, et al.* A voxel-based morphometric study of ageing in 465 normal adult human brains. *Neuroimage*. 2001; **14**(1 Pt 1): 21-36.

12. Seidlitz, J, Sponheim, C, Glen, D*, et al.* A population MRI brain template and analysis tools for the macaque. *Neuroimage*. 2018; **170**: 121-31.

13. Jenkinson, M, Smith, S. A global optimisation method for robust affine registration of brain images. *Medical image analysis*. 2001; **5**(2): 143-56.

14. Andersson, J, Jenkinson, M, Smith, P*, et al.* Non-linear registration, aka Spatial normalisation. FMRIB technical report TR07JA2. 2007.

15. Smith, SM, Jenkinson, M, Johansen-Berg, H*, et al.* Tract-based spatial statistics: voxelwise analysis of multi-subject diffusion data. *Neuroimage*. 2006; **31**(4): 1487-505.

16. Jenkinson, M, Beckmann, CF, Behrens, TE*, et al.* FSL. *Neuroimage*. 2012; **62**(2): 782-90.

17. Andersson, JL, Skare, S, Ashburner, J. How to correct susceptibility distortions in spin-echo echo-planar images: application to diffusion tensor imaging. *Neuroimage*. 2003; **20**(2): 870-88.

18. Calabrese, E, Badea, A, Coe, CL*, et al.* A diffusion tensor MRI atlas of the postmortem rhesus macaque brain. *Neuroimage*. 2015; **117**: 408-16.

19. Reveley, C, Gruslys, A, Ye, FQ*, et al.* Three-Dimensional Digital Template Atlas of the Macaque Brain. *Cerebral cortex (New York, NY : 1991)*. 2017; **27**(9): 4463-77.

20. Jung, B, Taylor, PA, Seidlitz, J*, et al.* A comprehensive macaque fMRI pipeline and hierarchical atlas. *Neuroimage*. 2021; **235**: 117997.

21. Hartig, R, Glen, D, Jung, B*, et al.* The Subcortical Atlas of the Rhesus Macaque (SARM) for neuroimaging. *NeuroImage*. 2021; **235**: 117996.

22. Golub, MS, Hogrefe, CE. Fluoxetine: juvenile pharmacokinetics in a nonhuman primate model. *Psychopharmacology (Berl)*. 2014; **231**(20): 4041-7.

23. Anderson, GM. Peripheral and central neurochemical effects of the selective serotonin reuptake inhibitors (SSRIs) in humans and nonhuman primates: assessing bioeffect and mechanisms of action. *International journal of developmental neuroscience : the official journal of the International Society for Developmental Neuroscience*. 2004; **22**(5-6): 397-404.

24. Clarke, AS, Kraemer, GW, Kupfer, DJ. Effects of rearing condition on HPA axis response to fluoxetine and desipramine treatment over repeated social separations in young rhesus monkeys. *Psychiatry research*. 1998; **79**(2): 91-104.

25. Clarke, AS, Ebert, MH, Schmidt, DE*, et al.* Biogenic amine activity in response to fluoxetine and desipramine in differentially reared rhesus monkeys. *Biol Psychiatry*. 1999; **46**(2): 221-8.

26. Fontenot, MB, Padgett, EE, 3rd, Dupuy, AM*, et al.* The effects of fluoxetine and buspirone on self-injurious and stereotypic behavior in adult male rhesus macaques. *Comp Med*. 2005; **55**(1): 67-74.

27. Fontenot, MB, Musso, MW, McFatter, RM*, et al.* Dose-finding study of fluoxetine and venlafaxine for the treatment of self-injurious and stereotypic behavior in rhesus macaques (Macaca mulatta). *Journal of the American Association for Laboratory Animal Science : JAALAS*. 2009; **48**(2): 176-84.

28. Sawyer, EK, Howell, LL. Pharmacokinetics of fluoxetine in rhesus macaques following multiple routes of administration. *Pharmacology*. 2011; **88**(1-2): 44-9.

29. Shrestha, SS, Nelson, EE, Liow, JS*, et al.* Fluoxetine administered to juvenile monkeys: effects on the serotonin transporter and behavior. *Am J Psychiatry*. 2014; **171**(3): 323-31.

30. Golub, MS, Hogrefe, CE, Bulleri, AM. Regulation of emotional response in juvenile monkeys treated with fluoxetine: MAOA interactions. *European neuropsychopharmacology : the journal of the European College of Neuropsychopharmacology*. 2016; **26**(12): 1920-9.

**Supplementary Figures**


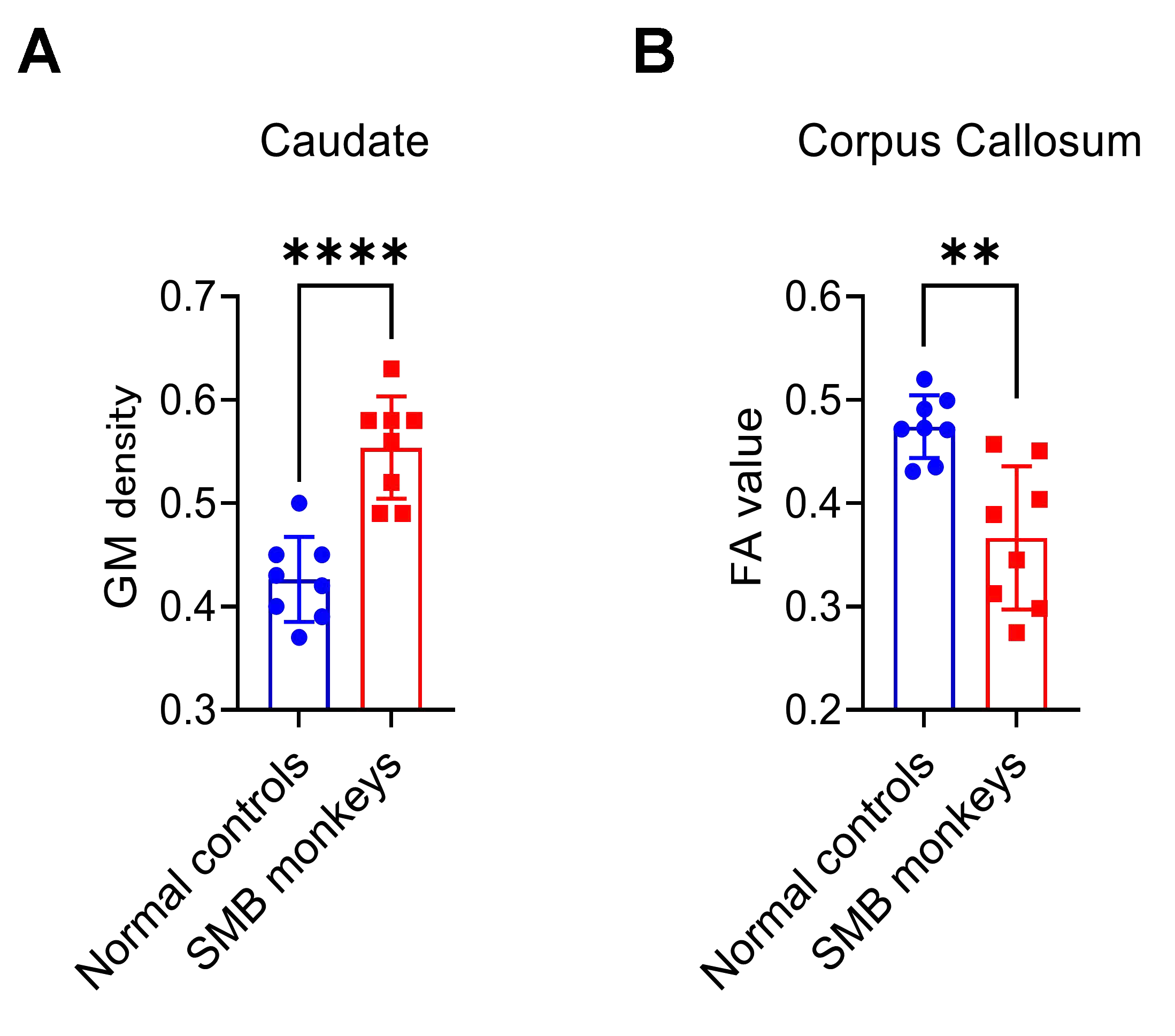


**Figure S1.** Gray matter (GM) density and fractional anisotropy (FA) value changes in monkey with SMBs. (A) Comparison of average GM volumes between SMBs monkeys and normal controls in the significant region (the left caudate nucleus) identified through VBM analysis. (B) Comparison of average FA values between SMBs monkeys and normal controls in the significant region (corpus callosum) identified through TBSS analysis. The data are expressed as mean ± SEM, and a two tailed *t*-test was used to compare 8 SMB monkeys against 8 normal controls. *P < 0.05; **P < 0.01; ***P < 0.001; ****P < 0.0001; ns represents no significant difference.


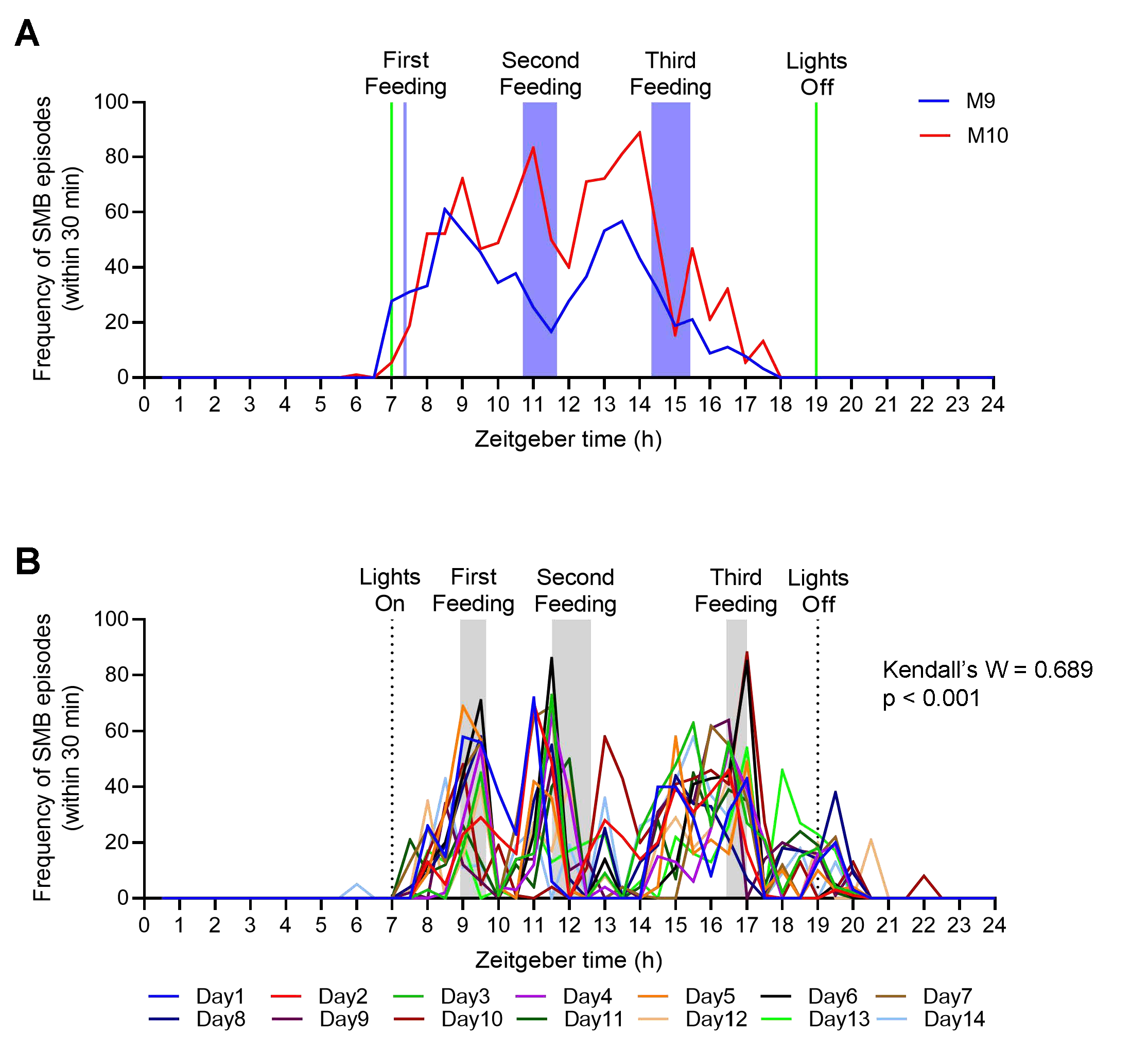


**Figure S2.** (A) The SMB frequency distribution pattern over a 24-hour period (n = 2). The frequency refers to the total number of SMB episodes during a 30-min interval. (B) The 24-hour distribution pattern of SMB frequency for monkey M2 over the course of 14 consecutive days. To assess the consistency of SMBs over the 14-day period, Kendall's W test was conducted.


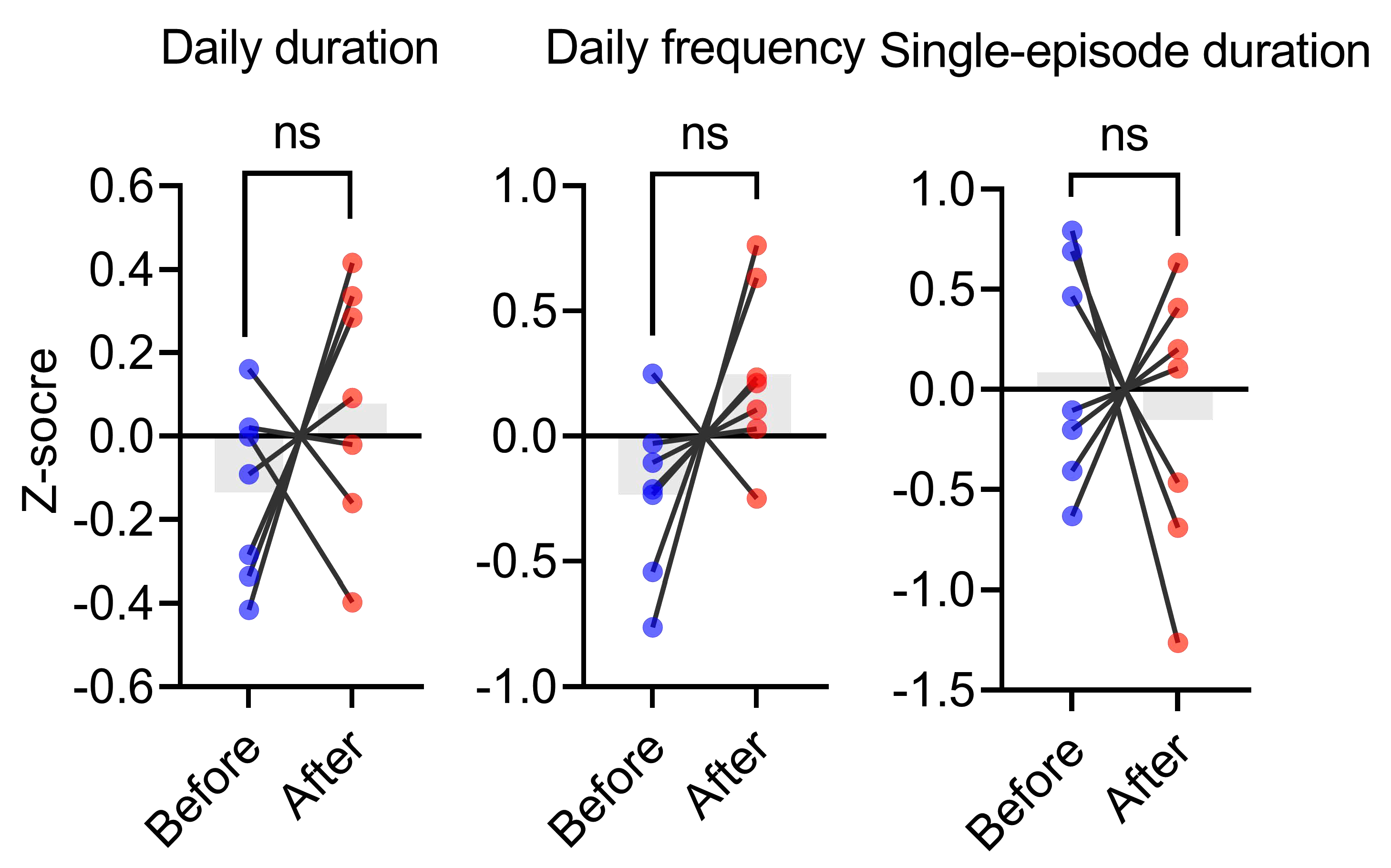


**Figure S3.** The effect of vehicle treatment on SMB episodes in monkeys. Z-scores of the total daily duration, frequency, and average single-episode duration of SMBs before and after treatment in vehicle-treated monkeys (n = 7). The SMB data were transformed using z-scores. *P < 0.05; **P < 0.01; ***P < 0.001; ****P < 0.0001; ns represents no significant difference.

**Supplementary Tables**

**Table S1**

| **Gene list** | **Author and year (reference)** |
| --- | --- |
| ASXL3 | Lin, G.N., et al. (2022) |
| HLA-A | Lin, G.N., et al. (2022) |
| PRPF38B | Lin, G.N., et al. (2022) |
| SLC35F5 | Lin, G.N., et al. (2022) |
| C19orf44 | Lin, G.N., et al. (2022) |
| ZNF77 | Lin, G.N., et al. (2022) |
| APEH | Lin, G.N., et al. (2022) |
| BRCA2 | Lin, G.N., et al. (2022) |
| CCNB1IP1 | Lin, G.N., et al. (2022) |
| GABRB1 | Lin, G.N., et al. (2022) |
| KCNK16 | Lin, G.N., et al. (2022) |
| SGSM1 | Lin, G.N., et al. (2022) |
| SPAG17 | Lin, G.N., et al. (2022) |
| MAGED1 | Lin, G.N., et al. (2022) |
| KDM3B | Lin, G.N., et al. (2022) |
| MAPK7 | Lin, G.N., et al. (2022) |
| AGO3 | Lin, G.N., et al. (2022) |
| U2SURP | Lin, G.N., et al. (2022) |
| SETD5 | Lin, G.N., et al. (2022) |
| TOP3A | Lin, G.N., et al. (2022) |
| ZNF418 | Lin, G.N., et al. (2022) |
| IKBKE | Lin, G.N., et al. (2022) |
| INPP4B | Lin, G.N., et al. (2022) |
| ASAP1 | Lin, G.N., et al. (2022) |
| DYSF | Lin, G.N., et al. (2022) |
| ELK3 | Lin, G.N., et al. (2022) |
| LMO7 | Lin, G.N., et al. (2022) |
| SCN5A | Lin, G.N., et al. (2022) |
| TRPC4AP | Lin, G.N., et al. (2022) |
| TENM1 | Lin, G.N., et al. (2022) |
| INTS5 | Lin, G.N., et al. (2022) |
| RPAP1 | Lin, G.N., et al. (2022) |
| NFATC3 | Lin, G.N., et al. (2022) |
| PPT2 | Lin, G.N., et al. (2022) |
| PPP4R3A | Lin, G.N., et al. (2022) |
| DNAJC7 | Lin, G.N., et al. (2022) |
| ZPLD1 | Lin, G.N., et al. (2022) |
| PRKD2 | Lin, G.N., et al. (2022) |
| KMT2B | Lin, G.N., et al. (2022) |
| ZNF471 | Lin, G.N., et al. (2022) |
| SLC8B1 | Lin, G.N., et al. (2022) |
| XRCC5 | Lin, G.N., et al. (2022) |
| OR5C1 | Lin, G.N., et al. (2022) |
| AKAP12 | Lin, G.N., et al. (2022) |
| SALL3 | Lin, G.N., et al. (2022) |
| LRCH2 | Lin, G.N., et al. (2022) |
| CHD8 | Lin, G.N., et al. (2022) |
| SCUBE1 | Lin, G.N., et al. (2022) |
| JPH4 | Mattheisen, M., et al. (2015) |
| PPP1R16 | Mattheisen, M., et al. (2015) |
| SORBS1 | Mattheisen, M., et al. (2015) |
| MEIS2 | Mattheisen, M., et al. (2015) |
| SLIT3 | Mattheisen, M., et al. (2015) |
| NSMCE2 | Mattheisen, M., et al. (2015) |
| DCC | Mattheisen, M., et al. (2015) |
| EYA4 | Mattheisen, M., et al. (2015) |
| CUBN | Mattheisen, M., et al. (2015) |
| ZFP64 | Mattheisen, M., et al. (2015) |
| PEX5L | Mattheisen, M., et al. (2015) |
| SEMA4D | Mattheisen, M., et al. (2015) |
| KIF16B | Mattheisen, M., et al. (2015) |
| C1orf26 | Mattheisen, M., et al. (2015) |
| PTPRD | Mattheisen, M., et al. (2015) |
| CDH9 | Mattheisen, M., et al. (2015) |
| CDH10 | Mattheisen, M., et al. (2015) |
| GRIK2 | Mattheisen, M., et al. (2015) |
| NEUROD6 | Mattheisen, M., et al. (2015) |
| SV2A | Mattheisen, M., et al. (2015) |
| GRIA4 | Mattheisen, M., et al. (2015) |
| SLC1A2 | Mattheisen, M., et al. (2015) |
| IQCK | Mattheisen, M., et al. (2015) |
| C16orf88 | Mattheisen, M., et al. (2015) |
| OFCC1 | Mattheisen, M., et al. (2015) |
| SLITRK3 | Mattheisen, M., et al. (2015) |
| CAMTA1 | Stewart, S.E., et al. (2013) |
| C10orf143 | Stewart, S.E., et al. (2013) |
| LDLRAD3 | Stewart, S.E., et al. (2013) |
| PKP2 | Stewart, S.E., et al. (2013) |
| LINC02395 | Stewart, S.E., et al. (2013) |
| KRT32 | Stewart, S.E., et al. (2013) |
| RNU2-32P | Stewart, S.E., et al. (2013) |
| COL1A1 | Stewart, S.E., et al. (2013) |
| TXNL1 | Stewart, S.E., et al. (2013) |
| FUT2 | Stewart, S.E., et al. (2013) |
| MAMSTR | Stewart, S.E., et al. (2013) |
| ARHGAP15 | Stewart, S.E., et al. (2013) |
| CYTIP | Stewart, S.E., et al. (2013) |
| RAI14 | Stewart, S.E., et al. (2013) |
| PDE4D | Stewart, S.E., et al. (2013) |
| EFNA5 | Stewart, S.E., et al. (2013) |
| NSG2 | Stewart, S.E., et al. (2013) |
| R3HDM2P2 | Stewart, S.E., et al. (2013) |
| NPM1P10 | Stewart, S.E., et al. (2013) |
| RPL5P21 | Stewart, S.E., et al. (2013) |
| ADCY8 | Stewart, S.E., et al. (2013) |
| ZBTB43 | Stewart, S.E., et al. (2013) |
| BTBD3 | Stewart, S.E., et al. (2013) |
| FAIM2 | Stewart, S.E., et al. (2013) |
| ISM1 | Stewart, S.E., et al. (2013) |
| DHRS11 | Stewart, S.E., et al. (2013) |
| GRIK4 | Stewart, S.E., et al. (2013) |
| GRIK1 | Stewart, S.E., et al. (2013) |
| SHANK3 | Stewart, S.E., et al. (2013) |
| ADARB2 | Stewart, S.E., et al. (2013) |
| ARHGAP18 | Stewart, S.E., et al. (2013) |
| JMJD2C | Stewart, S.E., et al. (2013) |
| DLGAP1 | Stewart, S.E., et al. (2013); Mattheisen, M., et al. (2015) |
| DLGAP3 | Stewart, S.E., et al. (2013); Mattheisen, M., et al. (2015) |
| SLC1A1 | Stewart, S.E., et al. (2013); Mattheisen, M., et al. (2015) |

**Table S2**

| **Mean diffusivity** | | |
| --- | --- | --- |
| Brain region (abbr.) | Full name | P-adj |
| ↓VPM-VPL # | ventroposterior medial and lateral thalamus | 0.002 |
| ↑PPit | posterior pituitary | 0.003 |
| ↓VPI # | ventroposterior inferior thalamus | 0.005 |
| ↑area_11 # | area 11 | 0.005 |
| ↓SpThal # | spinal thalamus | 0.007 |
| ↑OFC # | orbital frontal cortex | 0.008 |
| ↑area_11l # | lateral area 11 | 0.008 |
| ↓Ri | retroinsula | 0.008 |
| ↓LPul # | lateral pulvinar | 0.008 |
| ↓LVPal | lateral and ventral pallium | 0.009 |
| ↓ILThal # | intralaminar thalamus | 0.013 |
| ↓VThal # | ventral thalamus | 0.017 |
| ↓CbThal # | cerebellar thalamus | 0.018 |
| ↑area_11m # | medial area 11 | 0.019 |
| ↓VLPV # | ventral lateral posteroventral thalamus | 0.021 |
| ↓area_36c | caudal area 36 | 0.021 |
| ↓TEa | area TEa | 0.026 |
| ↑SDB | septum diagonal band | 0.029 |
| ↓VLPD # | ventral lateral posterodorsal thalamus | 0.029 |
| ↓APul # | anterior pulvinar | 0.029 |
| ↓CMn-PF # | centromedial-parafascicular thalamus | 0.031 |
| ↓Fl | fimbria | 0.033 |
| ↓PEa | area PEa | 0.034 |
| ↓VIP | ventral intraparietal area | 0.035 |
| ↓VMPo-VMB # | ventromedial posterior+basal thalamus | 0.037 |
| ↓Rt # | reticular_thalamus | 0.038 |
| ↓p1Rt | p1 mesencephalic reticulum | 0.038 |
| ↑EA | extended amygdala | 0.039 |
| ↓DStr # | dorsal striatum | 0.042 |
| ↓Str # | striatum | 0.043 |
| ↓Pons | pons | 0.043 |
| ↓LPal | lateral pallium | 0.044 |
| ↓Cl | claustrum | 0.046 |
| ↓VLA # | ventral lateral anterior thalamus | 0.046 |
| ↓VPons | ventral pons | 0.046 |
| ↓VLX # | ventral lateral x thalamus | 0.047 |
| ↑area_24a_prime # | area 24a prime | 0.049 |
| ↑area_10mr # | rostral medial frontal pole | 0.049 |
| ↓PNThal # | pallido nigral thalamus | 0.050 |
|  |  |  |
|  |  |  |
| 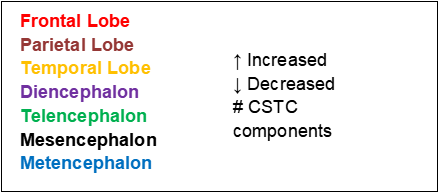 |  |  |
|  |  |  |
|  |  |  |
|  |  |  |
|  |  |  |
|  |  |  |
|  |  |  |
|  |  |  |

**Table S3**

| **ID: #** | **Gender** | **Age** | **Group** |
| --- | --- | --- | --- |
| M1 | Male | 7 | SMB monkey |
| M2 | Male | 4 | SMB monkey |
| M3 | Female | 4 | SMB monkey |
| M4 | Female | 6 | SMB monkey |
| M5 | Male | 4 | SMB monkey |
| M6 | Male | 4 | SMB monkey |
| M7 | Female | 3 | SMB monkey |
| M8 | Female | 3 | SMB monkey |
| C1 | Male | 9 | Control |
| C2 | Male | 9 | Control |
| C3 | Male | 9 | Control |
| C4 | Male | 9 | Control |
| C5 | Male | 9 | Control |
| C6 | Male | 6 | Control |
| C7 | Male | 9 | Control |
| C8 | Male | 8 | Control |
| C9 | Male | 8 | Control |
| C10 | Male | 10 | Control |
| C11 | Female | 6 | Control |
| C12 | Female | 9 | Control |
| C13 | Female | 10 | Control |
| C14 | Female | 9 | Control |
| C15 | Female | 9 | Control |
| C16 | Female | 14 | Control |
